# Digital remote maintenance inhaler adherence interventions in COPD: a systematic review and meta-analysis

**DOI:** 10.1183/16000617.0136-2024

**Published:** 2024-12-04

**Authors:** Hnin Aung, Ronnie Tan, Cara Flynn, Pip Divall, Adam Wright, Anna Murphy, Dominick Shaw, Tom J.C. Ward, Neil J. Greening

**Affiliations:** 1 Department of Respiratory Sciences, University of Leicester, Leicester, UK; 2 Institute for Lung Health, NIHR Respiratory Biomedical Research Centre, University Hospitals of Leicester, Leicester, UK; 3 School of Pharmacy, DeMontfort University, Leicester, UK; 4 Co-last authors

## Abstract

**Introduction:**

Sub-optimal inhaler adherence undermines the efficacy of pharmacotherapy in COPD. Digitalised care pathways are increasingly used to improve inhaler-use behaviour remotely. This review investigated the feasibility and impact of remote electronic inhaler adherence monitoring (EIM) and intervention platforms on clinical outcomes in COPD.

**Methods:**

A literature search was conducted and studies investigating maintenance inhaler use among people with COPD using digital technology were selected. Pairwise and proportional meta-analyses were employed with heterogeneity assessed using I^2^ statistics. When meta-analysis was not feasible, a narrative synthesis of outcomes was conducted.

**Results:**

We included 10 studies including 1432 people with COPD whose maintenance inhaler usage was supported by digital inhalers and apps featuring audiovisual reminders and educational content with or without engagement with healthcare providers (HCPs). Inhaler adherence rate (AR) varied with calculation methods, but an overall suboptimal adherence was observed among people with COPD. HCP-led adherence interventions alongside EIM improved mean AR by 18% (95% CI 9–27) *versus* passive EIM only. Enhanced AR may reduce COPD-related healthcare utilisation with little impact on health-related quality of life and exacerbation rate. Despite encountering technical issues among 14% (95% CI 5–23%) of participants, 85% (95% CI 76–94%) found digital platforms convenient to use, while 91% (95% CI 79–100%) perceived inhaler reminders as helpful.

**Conclusion:**

Digitalised interventions can enhance maintenance inhaler adherence in COPD but their overall effect on clinical outcomes remains uncertain. Further work is required to tailor interventions to individuals’ adherence behaviour and investigate their longer-term impact.

## Shareable abstract

**Electronic inhaler adherence monitoring can effectively identify sub-optimal adherence in people with COPD and potentially improve adherence when combined with healthcare professionals’ interventions. The impact on clinical outcomes remains uncertain.** https://bit.ly/3NfGtK5

## Introduction

Inhaled therapy is crucial for managing chronic airway disease, with approximately 630 million inhalers prescribed globally each year [1] and over 60 million used annually in the UK [2]. Despite its importance, sub-optimal adherence remains a significant challenge that hinders treatment effectiveness and optimal outcomes [3, 4]. Recognising and addressing nonadherence to inhaled therapy promptly can prevent unnecessary investigations or treatment escalation.

Inhaler-use behaviour among patients is highly individual, multifaceted and dynamic, necessitating repeated assessment over time [5]. Nonadherence, the extent to which patients’ actions do not align with the agreed recommendations [6], can be broadly classified as intentional and nonintentional [7]. Intentional nonadherence occurs when patients deliberately avoid following the treatment regime (*e.g.*, due to concerns regarding side-effects). Nonintentional nonadherence, more common in COPD [8], happens when patients are willing but unable to follow the treatment due to factors such as financial constraints or poor inhaler technique. Often, nonadherence categories overlap (*e.g.*, forgetfulness in taking inhalers may not be solely due to cognitive problems but also caused by a lack of motivation). Identifying these specific attributing factors to nonadherence is crucial before assigning an intervention.

Assessing inhaler adherence in COPD can be challenging due to variations in measurement methods. Conventional tools such as self-reporting scales [9–11], canister weighing and dose counting are limited by recall bias and the risk of dose dumping [12]. Prescription refill records do not guarantee real-time use of inhalers. On the other hand, the advancement of digital technology, accelerated by the coronavirus disease 2019 pandemic [13, 14], has led to an increased use of telemedicine in COPD care. Telemedicine, which involves the provision of clinical care through information and communication technologies [15], along with electronic medication monitors that can be integrated into different inhaler types [16], provides valuable insights into inhaler usage patterns and allows for timely interventions. These monitors provide information on the time of inhaler actuation, inspiratory flow technique, artificial intelligence-driven biofeedback and reminders. Despite their potential benefits, there are some concerns regarding their implementation costs, patients’ digital literacy and acceptance, and possible technical issues [17]. The effectiveness of these tools largely depends on addressing the specific reason for each patient's nonadherence and ensuring that the technology is accessible and acceptable to them.

Existing systematic reviews have focused on adherence to pharmacological therapy, but in combined asthma and COPD populations, with the former being the predominant study cohort [18, 19], or utilising nondigital assessment methods [3, 20, 21]. Some specifically addressed interventions for treatment adherence in COPD [22, 23], albeit the findings applied to a wide range of pharmacological therapies, including oral medications. To our knowledge, no systematic review has investigated the feasibility and impact of digitalised interventions on maintenance inhaler usage and clinical outcomes specific to the COPD population. This review aims to evaluate the feasibility and effectiveness of studies targeting maintenance inhaler adherence in COPD remotely using digital technology or telemedicine. The resultant impact on maintenance inhaler adherence rate (AR), health-related quality of life (HRQoL), exacerbation rate, COPD-related healthcare utilisation (HCU), inhaler technique and reliever use are examined. Furthermore, the role of interactions with healthcare providers (HCPs) in improving maintenance inhaler adherence is discussed.

## Methods

### Search strategy

A literature search was conducted on 12 April 2023 using the Medline, Cochrane Library, Embase and CINAHL databases with no restrictions on language and publication date. The review was prospectively registered in the International Prospective Register of Systematic Reviews (PROSPERO) (ID: CRD42023414344).

### Eligibility criteria

Eligible studies were selected using the following criteria:

- Type: randomised controlled trials (RCTs), cohort and proof-of-concept studies published in full text.
- Population: adult patients (≥18 years old) with a diagnosis of COPD, confirmed either clinically or *via* spirometry, who are using maintenance inhaler therapy. Studies that included multiple patient groups were considered eligible only if at least 90% of participants had COPD or results for the COPD subgroup were specifically presented.
- Intervention: remote intervention targeting maintenance inhaler usage *via* digital innovations such as digital inhalers and telemedicine, as the core component of the study. For RCTs, the comparator groups were required to receive either standard of care or alternative intervention methods meeting the aforementioned criteria.
- Outcomes: 1) maintenance inhaler use, including inhaler AR and techniques; 2) the impact of digitalised remote interventions on improving maintenance inhaler use and their effect on exacerbation rate, HRQoL, HCU and reliever usage; 3) the role of additional HCP involvement; and 4) feasibility and acceptability of the interventions included in the analysis.

### Data extraction and assessment of risk of bias

Results from the literature search were screened in Rayyan software based on titles and abstracts by two independent reviewers (H.A. and C.F.). Discrepancies were resolved through consultation with a third reviewer (R.T.). Data extraction was conducted using a customised Excel spreadsheet by H.A. and R.T. The risk of bias for each study was assessed using ROBINS-I (Risk Of Bias In Non-randomized Studies – of Interventions) [24] and RoB 2 (Risk of Bias 2) [25]. The quality of evidence and strength of recommendations were evaluated using the “Grades of Research, Assessment, Development and Evaluation” (GRADE) approach [26]. The summary of findings was presented *via* GRADEpro GDT (www.guidelinedevelopment.org) software.

### Statistical analysis

Changes in effect for continuous outcomes were presented *via* standardised mean differences (95% CI). Meta-analysis and proportional meta-analysis were performed *via* meta packages in R (version 4.3.1). Between-study heterogeneity was assessed using I^2^ and publication bias was assessed through visual inspection of the funnel plots [27]. Random-effect models were used to pool the observed treatment effect. Sensitivity analyses were not performed due to the limited number of studies.

## Results

### Study selection and population characteristics

**FIGURE 1:**
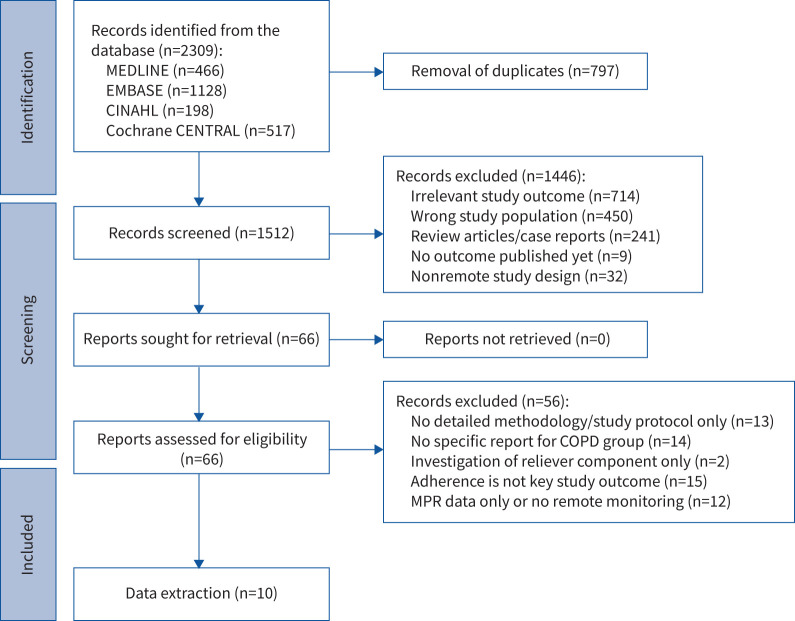
PRISMA flow diagram for the literature search and study selection. MPR: medication possession ratio.

Of 2309 articles identified through the search strategy, 2243 were excluded upon screening their titles and abstracts (see figure 1), leaving 66 reports for full-text reviews. Ultimately, 10 articles (three RCTs and seven cohort studies) met full inclusion/exclusion criteria, involving 1432 people with COPD, published between 2011 and 2022. Most studies were conducted in the USA (n=5) while the rest were in Ireland (n=1), the UK (n=2), Indonesia (n=1) and New Zealand (n=1). Recruitment primarily occurred in primary (n=5) and secondary (n=3) care settings with some from healthcare databases/social media platforms (n=2).

Meta-analysis revealed that participants were predominantly female, accounting for 61% (95% CI 59–64) across nine studies. However, one study [28] included exclusively male subjects, based in a centre serving veterans. The mean age was 67 years (95% CI 61.7–72.4) with a median follow-up period of 3.2 (interquartile range 1–6) months. As reported in seven studies, the mean number of COPD exacerbations/HCUs per participant in the prior year was 1.2 (95% CI 0.2–2.3). Five studies provided baseline lung function and ethnicity data, with a mean forced expiratory volume in 1 s of 40.3% (95% CI 27.5–53.1) predicted and 91% (95% CI 88–94) of participants were identified as of European descent. Participants’ education backgrounds were documented in only two studies and health literacy status was reported by one group, suggesting that these factors were less frequently addressed compared to other variables.

### Risk of bias within studies and quality assessment

This risk of bias assessment for RCTs revealed “some concerns to high” risk of bias in two of three studies due to limited participant blinding, as well as instances of missing outcomes and reports from participants who were unable to comply with study procedures. For cohort studies, varying degrees of risk of bias were observed as assessed by ROBINS-I, namely moderate (n=2) to serious (n=3) and critical (n=2), see supplementary figure A. A summary of the quality of evidence is presented in table S1.

### Review of study designs examining maintenance inhaler use electronically in COPD

Seven studies (five cohort studies and two RCTs) investigated maintenance inhaler adherence using electronic inhaler adherence monitoring (EIM). Digital devices were attached to participants’ inhalers to track inhaler usage in real time, with the data transferred to manufacturer-specific mobile apps *via* Bluetooth. Three studies [29–31] used the Propeller Health (Madison, USA) platform, two [32, 33] employed Inhaler Compliance Assessment (INCA^TM^) devices (Vitalograph, Ireland) and one [34] incorporated Adherium's Hailie^®^ and one implemented BreatheMate devices [35].

Five studies [29–31, 34, 35] incorporated supplementary digitalised adherence aids, such as tailored audiovisual inhaler reminders (either as pre-emptive, missed or overuse alerts), educational content regarding COPD and inhaler usage trends displayed within the apps. Of these, three [31, 34, 35] included interventions from HCPs in response to adherence alerts. In two studies [32, 33] that used INCA^TM^, inhaler usage was monitored passively without the aid of supplementary platforms. INCA^TM^ records a time-stamp and the quality of the inhalation technique by recording acoustic signatures. Two RCTs [34, 35] directly compared the benefits of passive EIM alone *versus* behavioural modification facilitated by HCPs through digital tools alongside EIM (see tables 1 and S1 for details of the user interfaces).

**TABLE 1:** Characteristics of studies included in the review

Study, year, sample size/follow-up period	IG	Major features and settings of the study apps	Additional support from HCPs	CG	Calculation of AR or inhaler technique	Key outcomes including AR to maintenance inhalers
**Randomised controlled trials**						
**Broadbent *et al.* [34], 2018**, **n=60/4 months**	iRobi robot-assisted self-management integrated with Adherium's Hailie® digital inhalers	Tracks the date and time of actuation with feedback on inhaler use trends and health status Scheduled reminders and education modules for inhaler and rehabilitation Measures oxygen level, FEV_1_ and HR	Adherence alerts (if medications were missed ≥3 consecutive times) Opens conversations with study physiotherapists on alerts	Passive EIM with no reminders/intervention	Percentage of doses taken as prescribed over 4 months Six-item MAR scale	*Primary outcome* No significant difference in number of respiratory admissions and days spent in the hospital ITT analysis (IG *versus* CG): 15 *versus* 15 in both groups, (p>0.99) and 50 *versus* 65 (p=0.90) *Secondary outcome* Mean±sd AR: 48.5±34.1% *versus* 29.5±32.4%, p=0.03, IG *versus* CG (electronic record) Mean±sd AR: 23.08±2.63 *versus* 22.27±4.12, p=0.04 IG *versus* CG (MAR scale) Mean±sd total CCQ scores: 16.3±6.3 *versus* 18.2±7.0, p=0.36, IG *versus* CG No difference in direct hospitalisation costs between the two groups, p=0.32
**Criner *et al.*** [**35], 2021**, **n=138/6 months**	BreatheMate digital inhalers with support from HCP	Tracks the date and time of actuation Pre-emptive as well as missed dose reminders Monthly reminders to obtain refills and overuse alerts	Direct contact from the study team with inhaler overuse alerts (*i.e.* >10 puffs·day^−1^ of controllers)	Passive EIM with no reminders/intervention	Percentage of adherent days (*i.e.* days with two sets of two puffs taken within 60 min of each other) AR ≥80% during the study period	*Primary outcome* Mean AR: 77.6% *versus* 60.2%; IG *versus* CG, p<0.001 Odds of AR ≥80%: 3.07 (95% CI 1.49–6.52), IG *versus* CG Days of overuse (>2 sets of 2 puffs·day^−1^), underuse (<2 sets of 2 puffs·day^−1^ and no use were lower in IG compared to CG, p<0.05 *Secondary outcome* Mean±sd CCQ scores: 2.18±0.82 *versus* 2.39±1.17, IG *versus* CG, p=0.80 *Others* Odds of AR ≥80% for age ≥65 years *versus* age <65 years: 4.6
**North *et al.*** [**37], 2020**, **n=41/90 days**	Multi-faceted online app (myCOPD) with weekly pre-recorded inhaler training videos	Educational information on COPD and treatment, pulmonary rehabilitation Videos explaining the correct technique for different inhaler devices and pulmonary rehabilitation Appointment diary and oxygen alert card 5-day local weather and pollution report	Not involved	Routine care with a written self-management action plan in the event of deterioration	Number of inhaler technique errors as per device-specific manufacturer guidelines	*Key outcomes* Continual decline in number of app users per week for 90 days MD of CAT score: −4.49 (95% CI −8.41–0.58), IG *versus* CG Number of technique errors, adjusted IRR: 0.38 (95% CI 0.18–0.80), IG *versus* CG Number of exacerbations, adjusted IRR: 0.58 (95% CI 0.32–1.04), IG *versus* CG Odds of readmission: 0.38 (95% CI 0.07–1.99), IG *versus* CG
**Observational studies**						
**Yawn *et al.*** [**30], 2021**, **n=122/6 months**	Propeller Health (USA) digital inhalers linked with mobile app	Tracks the date and time of actuation with feedback on inhaler use trends Access to education on COPD-related triggers on the app AV reminders if no maintenance inhaler uses for >4 consecutive days or above average use of reliever	Not applicable	Not applicable	Percentage of adherent days (*i.e.* days with correct sets of puffs taken)	*Primary outcome* Mean (%) of days using app: 49.5 (31) *Secondary outcome* Mean±sd AR: 77.4±21.5% Mean±sd CAT score: pre 21±7.4 *versus* post 20.2±8.3 Mean±sd reliever use: 1.3±3 puffs·day^−1^ Number of reliever-free days: 58.3±24.4 days within 24 weeks
**Alshabani *et al.*** [**31], 2020**, **n=39/1 year**	Propeller Health (USA) digital inhalers linked with mobile app	Tracks the date and time of actuation with feedback on inhaler use trends AV reminders if no maintenance inhaler use for >4 consecutive days or reliever use ≥1.64 times (sd) above average	Opens conversations with HCPs	Not applicable	Percentage of prescribed doses taken, truncated at 100% for each day	*Primary outcome* All-cause HCU per year: pre 3.4 (sd 2.6) to post 4.7 (sd 4.1), p=0.06 *Secondary outcome* Mean AR (adjusted with hospitalisation or ED visits): 46.2%, declined by 0.46% per week, p<0.0001 COPD-related HCU per year: pre 3.4 (sd 3.2) to post 2.2 (sd 2.3), p=0.01
**Kaye *et al.*** [**29], 2021**, **n=663/90 days**	Propeller Health (USA) digital inhalers linked with mobile app	Tracks the date and time of actuation with feedback on inhaler use trends Scheduled app-based inhaler reminders Educational content on COPD Additional gamified features if adherence resistant to all app-derived measures	Not involved	Not applicable	Percentage of prescribed doses taken, truncated at 100% for each day	*Primary outcome* Odds ratio of maintenance inhaler use: 1.61 (95% CI 1.49–1.75) opening *versus* not opening app *Secondary outcome* Maintenance inhaler AR 62% (sd 32) Participants aged 40–60 years had greater odds of using maintenance inhalers than those >60 years
**Sulaiman** ***et al.*** [**32], 2017**, **n=244/1 month**	INCA^TM^ attached to salmeterol/fluticasone DPIs	Collects acoustic recordings of inhalations and provides the date, time and quality of inhaler actuation	Not involved	Not applicable	Attempted AR (%) of doses taken at correct intervals Actual AR (attempted AR with no technique errors)	*Key outcomes* Mean±sd attempted AR: 59.8±29.5%; extra doses: 10.9±12.5%; missed doses: 37.6±26.4% Mean±sd actual AR: 22.7±29.3% 6% had actual AR >80% Mean±sd inhaler technique errors: 20.7±18.3 Poor lung function distinguished those with poor adherence and frequent technique errors
**Hesso *et al.*** [**33], 2020**, **n=29/1 month**	INCA^TM^ attached to salmeterol/fluticasone DPIs	Collects acoustic recordings of inhalations and provides the date, time and quality of inhaler actuation	Not involved	Not applicable	Attempted AR (%) of doses taken at correct intervals Actual AR (attempted AR with no technique errors)	*Key outcomes* Median (IQR) attempted AR: 78.8% (17.1–100) Actual AR: 47.4% (0–93) 17% had actual AR ≥80% Mean inhaler technique errors: 12 (95% CI; 8–29) Median (IQR) from dose counter (% of PDC) and medication refill: 100 (76.7–100) and 100 (50–133), respectively
**Locke *et al.*** [**28], 2019**, **n=74/3 months**	Remote inhaler training	Software (Cisco Jabber Video for Telepresence 4.5) and a webcam provided to patients	Monthly inhaler training from study pharmacist over videoconference calls	Not applicable	Inhaler technique errors based on TTG scores	*Key outcomes* Improved inhaler technique at 2 months across nearly all inhalers (p<0.01), except ipratropium pMDI users No significant difference in ED visits and hospitalisations pre- and post-intervention
**Sauriasari *et al.*** [**36], 2021**, **n=22/3 months**	Pre-recorded online training sessions sent *via* WhatsApp	Pre-recorded online training video (<2 min) detailing inhalation technique	Not involved	Not applicable	Inhaler technique errors using a device-specific checklist	*Key outcomes* 63.6% of patients showed persistent technique errors DPI type was most used improperly (62.5%) Mean CAT score changed by −2 (12.8±1.3 *versus* 10.8 ±2.0) pre- and post-intervention 68.2% had a decrease of CAT score ≥2

AR: adherence rate; AV: audiovisual; CAT: COPD Assessment Tool; CCQ: Clinical COPD Questionnaire; CG: comparator group; DPI: dry powder inhaler; ED: emergency department; EIM: electronic inhaler adherence monitoring; FEV_1_: forced expiratory volume in 1 s; HCP: healthcare provider; HCU: healthcare utilisation; HR: heart rate; IG: intervention group; INCA^TM^: Inhaler Compliance Assessment; IQR: interquartile range; IRR: incidence rate ratio; ITT: intention to treat; MAR: Medication Adherence Report; MD: mean difference; pMDI: pressurised metered dose inhaler; PDC: proportion of days covered; TTG: teach-to-goal.

### Digital assessment of maintenance inhaler AR among people with COPD using EIM based on different calculation methods

**FIGURE 2:**
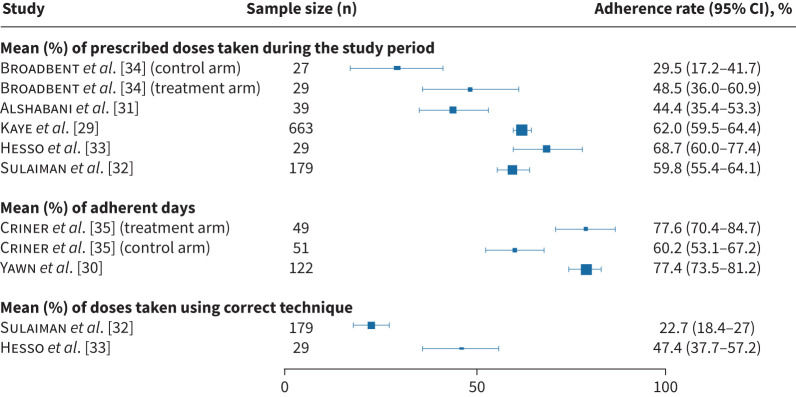
Maintenance inhaler adherence rate (AR) among people with COPD using electronic inhaler adherence monitoring (EIM) as per different calculation methods, as follows: mean (%) of prescribed inhaled doses taken during the study period, mean (%) of adherent days (*i.e.* days with correct sets of inhalations) and mean complete AR (%) of inhaled doses taken with proper timing, intervals and technique assessed by the area under curve metric.

The methods for calculating maintenance inhaler AR varied between studies, as shown in the forest plot (see figure 2). These include:

1) Percentage of prescribed inhaled doses taken during the study period: the most common method, used in five studies [29, 31–33, 34], truncating to 100% for each day.
2) Percentage of adherent days: defined as days with correct sets of puffs (*e.g.*, two sets of two puffs taken within 60 min of each other for a twice-daily regimen).
3) Complete AR: only the inhaled doses taken correctly regarding method, interval, and timing were extracted using acoustic recordings from the INCA^TM^ in two studies. In the first study, 69% of participants had periods of inhaler overuse, 92% had missed doses and only 8% showed no period of missed or extra dosing within a month. In the second study, the mean percentages for excessive and missing doses were 10.9% (sd 12.5) and 37.6% (sd 26.4), respectively.

Three studies set the target AR threshold as ≥80%. However, a low proportion of participants achieved the mean complete AR of ≥80%. Specifically, in adherence assessment using INCA^TM^ technology, only 17% (n=5/29) [32] and 6% (n=11/179) [33] met the threshold.

### Digital assessment of inhalation technique errors among people with COPD

Three studies reported inhalation technique errors among people with COPD. Hesso *et al.* [33] and Sulaiman *et al.* [32] employed INCA^TM^ devices. The former identified multiple inhalations (49.5%) and drug priming without subsequent inhalation (29.5%) as the most common errors among 29 participants. Meanwhile, the latter found that insufficient inspiratory flow rate (<35 L·min^−1^) (47.9%) and multiple inhalations (23%) were most prevalent among 244 participants. Sauriasari *et al.* [36] assessed inhaler techniques using a device-specific checklist. Among pressurised metered dose inhaler (pMDI) users, the primary error occurred during inhaler removal from the mouth while holding breath (25%) and, for dry powder inhaler users, it was the failure to exhale gently away from the device before inhalation (83%).

### The impact of digitalised maintenance inhaler adherence intervention on COPD-related HCU and exacerbation rate

Three studies investigated the association of maintenance inhaler adherence intervention with COPD-related HCU and exacerbation rate. However, variations in outcome reporting methods precluded meta-analysis. In an RCT led by North *et al.* [37], the treatment group receiving virtual inhaler technique training demonstrated a reduced exacerbation rate ratio of 0.58 (95% CI 0.32–1.04) and a lower likelihood of readmission (odds ratio 0.38, 95% CI 0.07–1.99) compared to the control group. Conversely, Locke *et al.*’s [28] cohort study found no significant change in the exacerbation rate post-intervention. Similarly, all-cause emergency department visits and hospitalisations were not significantly affected, although numerical results were not provided.

Alshabani *et al.* [31] observed a decreased mean COPD-related HCU per participant per year, pre 3.4 (sd 3.2) *versus* post 2.2 (sd 2.3), p=0.01, following digitalised maintenance inhaler adherence support, but no significant difference in mean rescue pack usage, pre 1.9 (sd 1.9) *versus* post 1.8 (sd 2.4), p=0.34. Overall, very low-certainty evidence suggested that digitalised inhaler adherence interventions may improve COPD-related HCU but have minimal influence on the exacerbation rate, see table S2.

### The impact of digitalised maintenance inhaler adherence intervention on HRQoL in COPD

**FIGURE 3:**
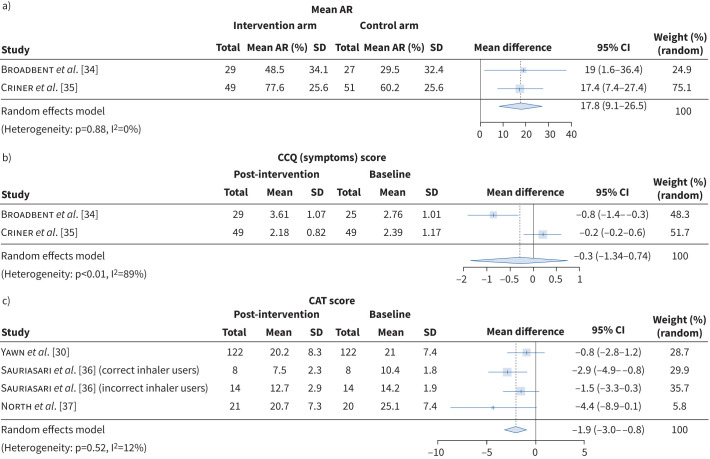
Change in a) mean maintenance inhaler adherence rate (AR) and b) Clinical COPD Questionnaire (CCQ) scores between digitalised interventions delivered by healthcare providers with full features *versus* standalone passive electronic inhaler adherence monitoring (EIM) among people with COPD. c) Overall change in COPD Assessment Test (CAT) scores by digitalised interventions. The funnel plots are provided in figures B and C of the supplementary file.

Meta-analysis of outcomes from three studies [30, 36, 37] investigating HRQoL demonstrated an improvement in COPD Assessment Test scores by −1.9 (95% CI −3.0–−0.8) with enhanced maintenance inhaler use, but did not reach a minimal clinically important difference (MCID), see figure 3c.

One study assessing the effect of inhaler technique support found a mean change of −1.48 (95% CI −7.82–4.86) in participants’ St George's Respiratory Questionnaire scores that did not reach the MCID [38]. Similarly, meta-analysis results from two RCTs [34, 35] showed a mean improvement of the Clinical COPD Questionnaire (CCQ) score by −0.3 (95% CI −1.3–0.7) (see figure 3b**)** below the MCID threshold of 0.4 [39]. Thus, low-certainty evidence suggests that digitalised adherence interventions may not improve HRQoL.

### Role of HCPs in digitalised inhaler adherence support in COPD

Meta-analysis of the results from two RCTs [34, 35] suggested an increase in mean maintenance inhaler AR by 17.8% (95% CI 9.1–26.5) in the treatment group receiving the full features of the digital platform along with telephone consultations with HCPs. This was compared to the control group supported solely by EIM without extra app features, see figure 3a. As described above, no significant change in CCQ scores was observed in both studies. Based on this, moderate certainty evidence indicates that HCP-led digitalised adherence interventions may enhance inhaler adherence compared to passive EIM.

### Effectiveness of digitalised intervention strategies in improving inhaler techniques among people with COPD

Three studies (one RCT and two cohort studies) evaluated the feasibility and effectiveness of digitalised strategies in improving inhaler device handling among people with COPD. An RCT by North *et al.* [37] compared the efficacy of weekly pre-recorded training videos *via* the multi-component (myCOPD) app against written self-management action plans including inhaler use instructions. The study found that using the app reduced the incident rate ratio of technique errors to 0.38 (95% CI 0.18–0.80) at day 90.

For cohort studies, monthly live coaching sessions by study pharmacists from Locke *et al.* [28] for 3 months led to a significant and sustained improvement in the participants’ inhaler technique across most inhaler devices (p<0.01), except for ipratropium pMDIs. The teach-to-goal approach was adopted, entailing repeated training until participants achieved mastery of the correct inhaler technique. Meanwhile, Sauriasari *et al.* [36] combined initial in-person demonstrations of inhaler use with brief pre-recorded videos (<2 min long) sent *via* WhatsApp once later within 3 months. Despite this, 63.6% (n= 14/22) persistently showed inhaler technique errors at the study end-point.

### The impact of electronic maintenance inhaler adherence intervention on reliever use in COPD

Two studies investigated the use of relievers (*i.e.*, short-acting beta agonists) and maintenance inhalers electronically among people with COPD. Yawn *et al.* [30] observed mean reliever use of 1.3 (sd 3.0) daily and 58.3 (sd 24.4) reliever-free days over 24 weeks, with an increasing trend of reliever-free days towards the end of the study. In Alshabani *et al.* [31], participants consumed a mean of 0.8 (sd 0.7) reliever puffs daily.

### Feasibility and acceptability of digitalised maintenance inhaler adherence interventions among people with COPD and healthcare professionals

**FIGURE 4:**
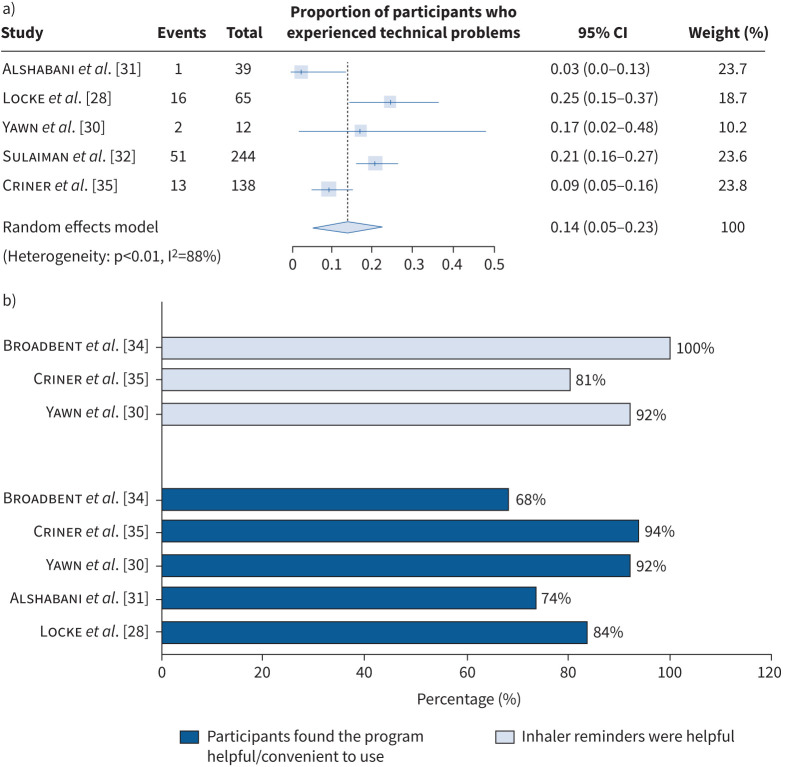
a) Proportion of participants who experienced technical problems during study participation. b) Feedback on platform usage and its inhaler reminders provided by participants who completed digitalised interventions on maintenance inhaler use.

Meta-analyses of participant feedback surveys demonstrated a total attrition rate of 18% (95% CI 10–27%) and 14% (95% CI 5–23%) experienced issues of lost/malfunctioning study devices or smartphone apps during their participation. Despite this, 85% (95% CI 76–94%) found the study platforms easy to follow and 91% (95% CI 79–100%) perceived inhaler reminders generated by the platforms as helpful, see figure 4. Details of the meta-analysis are provided in supplementary figures D–G. In the virtual inhaler training survey conducted by Locke *et al.* [28], 95.5% of participants preferred videoconferencing over in-person visits and 75.8% believed that they would not receive inhaler coaching if they had waited for a face-to-face appointment.

On the other hand, healthcare professionals from Locke *et al.* [28] encountered technical issues in 59.6% of visits (130/218), lasting an average of 8.4 (11.5) min per visit. In the study by Broadbent *et al.* [34], 50% of study robots had hardware problems, requiring 62 h in total of troubleshooting. Criner *et al.* [35] also recorded that 17% of participants’ smartphones were not synchronised with the study database for over 28 days at one point, limiting timely data transfer. Nonetheless, the majority of the study team responded that the research web portal was easy to use, see figure 4.

## Discussion

Our systematic review evaluated various digital strategies to improve maintenance inhaler use among individuals with COPD. EIM uncovered suboptimal adherence to maintenance inhalers. Studies employing scheduled reminders missed dosing alerts and subsequent consultations with HCPs showed a higher AR. However, participants were still nonadherent on 30% of study days. While digitalised adherence aids improved maintenance inhaler adherence and reduced COPD-related HCU, there was no significant improvement in HRQoL or exacerbation rate.

Although a direct comparison of digitalised adherence behavioural support involving HCPs against passive EIM standalone in two RCTs showed an additional adherence boost, the specific role of HCPs remains unclear. Participants received inhaler reminders accompanied by app-based education, with HCPs involved only when study-defined thresholds for missed or excessive dosing were met. However, the frameworks of behavioural intervention were not provided. It is uncertain whether the observed benefits were primarily due to interaction with HCPs, exposure to full features of digital platforms or both. Importantly, no significant additional effect on clinical outcomes resulted. Our findings support the existing evidence that interventions targeting maintenance inhaler adherence have led to only modest increases in adherence without drastically altering clinical outcomes [23, 40, 41].

On the other hand, there remains little consensus on setting up digitalised adherence support models. In RCTs, control groups were unblinded to the treatment allocation and equipped with study digital inhalers, potentially introducing the “Hawthorne effect” [42] and masking the true treatment effect. Moreover, all studies that reported power calculations failed to meet their recruitment target.

Given the limited net benefit in clinical outcomes and a small baseline mean exacerbation rate per participant (*i.e.*, 1.23 (95% CI 0.18–2.27), digitalised intervention studies require a large sample size to yield meaningful impacts. Furthermore, telemedicine-based programmes focusing on repeated inhaler training demonstrated favourable improvement in inhaler technique, contrary to those delivering remote education only once. This supports existing knowledge that repeated interventions increase the likelihood of achieving effective inhalation skills in chronic airway disease [43, 44].

While telemedicine may help capture the patients’ inhaler use, a one-size-fits-all approach to inhaler adherence intervention is insufficient due to the heterogeneity of behavioural traits among COPD patients. Further considerations should include individuals’ illness perceptions, expectations, capacity and concerns regarding prescribed therapy [45]. Challenges on the clinician's end such as delayed recognition of nonadherence and time constraints to explore patients’ perspectives [46] need to be addressed. Another important aspect is the role of biological endotypes in enhancing therapeutic decisions in COPD. Previous evidence [47, 48] confirmed the significance of type-2 inflammatory biomarkers, showing a strong correlation between inhaled corticosteroid (ICS) responsiveness and blood eosinophil count [47, 49–51] Considering this, adherence to ICS may benefit individuals with eosinophilic inflammation compared to neutrophilic inflammation (*e.g.*, WISDOM and SUNSET studies [52, 53]). Several studies in asthma have applied this sequential relationship between adherence and biomarkers (*e.g.*, the utility of fractional exhaled nitric oxide in identifying prior nonadherence and ICS sensitivity [54–57]). Thus, future research on personalised inhaler adherence support in COPD should target adherence behaviour and disease phenotypes.

### Proposal for future study designs

**FIGURE 5:**
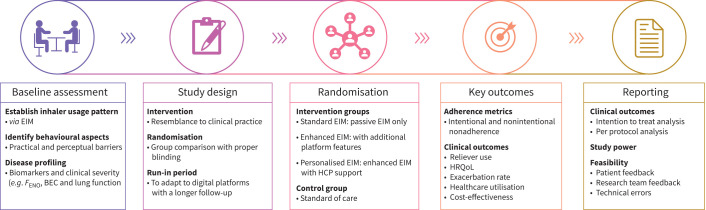
Key elements to consider for future study designs. BEC: blood eosinophil count; EIM: electronic inhaler adherence monitoring; *F*_ENO_: fractional exhaled nitric oxide; HCP: healthcare practitioner; HRQoL: health-related quality of life.

Herein, we suggest the following aspects to consider when designing a study to improve maintenance inhaler adherence and assess its impact on patients with COPD (figure 5). This approach integrates digital technology to support adherence while ensuring real-world applicability.

#### Baseline assessment

1) Characterisation of inhaler use: establish baseline inhaler usage patterns through passive EIM.
2) Behavioural insights: identify individual perceptual barriers to adherence (*e.g.*, structured interviews).
3) Disease profiling: assess biomarkers and clinical severity (*e.g.*, blood eosinophil count and lung function).

#### Study design

1) Group comparison: implement randomised group comparisons with proper blinding and maximal resemblance to clinical practice.
2) Duration: longer study period to assess the sustainable impact, preferably 12 months.
3) Mitigation of the Hawthorne effect [42]: allow a pre-study run-in period for patients to adapt to digital platforms and a wash-out period for previous inhalers.

#### Randomised interventions

Intervention groups:

1) Standard EIM: passive EIM only.
2) Enhanced EIM: passive EIM with digital aids such as audiovisual reminders, usage feedback and interactive coaching.
3) Personalised support: enhanced EIM with personalised intervention sessions from HCPs.

Control group: standard of care with no additional intervention.

#### Outcomes and reporting

1) Outcome measures: adherence metrics covering both intentional and nonintentional nonadherence, clinical outcomes including HRQoL, exacerbation rate, HCU and cost-benefit analysis where possible.
2) Feasibility and perspectives: feedback from both patients and research teams on the practicality and effectiveness of interventions to inform future resource allocation.
3) Reporting: study power, recruitment outcomes, conduct intention to treat and per protocol analyses.

### Limitations

The review has several limitations. Adherence outcomes vary with different calculation methods, emphasising the need for a standardised approach. Participant selection favoured those proficient with bespoke study equipment thereby limiting the generalisability of findings. Technical issues also led to the exclusion of some participants from the final analysis and limited follow-up periods underscore further exploration into long-term benefits and behavioural changes. The optimal levels of technology assistance in the COPD group remain undetermined as participants’ compliance with study digital tasks often hinges on their prior experience of using technology. Our findings call for larger studies comparing more diverse telehealth inhaler adherence interventions with different digital packages. In addition, adherence outcomes in this review predominantly relied on inhalation timestamps captured by electronic inhalation monitors. While newer generations devices [58] offer more advanced measurements, such as inspiratory effort [21, 58–62], their efficacy evaluations are clearer in asthma than in COPD. Therefore, stakeholders’ input is crucial to launching readily accessible digital inhalers capable of capturing inhalation physiology in the COPD population.

Overall, digitalised interventions were well-received by research practitioners and participants, offering a more comprehensive characterisation of patients’ inhaler use patterns compared to traditional methods. Feasibility outcomes also suggest that digital tools could expand access to inhaler adherence support, especially in rural areas where resources may be scarce. Additionally, the increasing integration of EIM into chronic airway disease research suggests an impending transformation towards establishing it as the “new normal” in comprehending and managing patients’ behaviour in future COPD studies.

## Conclusion

This review offers an insight into the evolving landscape of digital inhaler health platforms in COPD, highlighting end-user acceptability and their effectiveness. While digital platforms show promise in enhancing patient–professional engagement, suboptimal adherence to maintenance inhalers remains prominent among people with COPD. Future clinical trials on digitalised adherence intervention should improve the generalisability and simplification of user interfaces while considering multi-dimensional perspectives from patients, clinicians and underlying biological mechanisms.

## Supplementary material

10.1183/16000617.0136-2024.Supp1 **Please note:** supplementary material is not edited by the Editorial Office, and is uploaded as it has been supplied by the author.
Supplementary material ERR-0136-2024.SUPPLEMENT
