# Supplementary material for: Digital remote maintenance inhaler adherence interventions in COPD: a systematic review and meta-analysis
Source: Eur Respir Rev. 2024 Dec 4;33(174):240136. doi: 10.1183/16000617.0136-2024 (PMC11615661; doi:10.1183/16000617.0136-2024)
Supplement: Supplementary file 1 [file ERR-0136-2024.SUPPLEMENT.pdf]

Digital remote maintenance inhaler adherence interventions in Chronic Obstructive Pulmonary Disease (COPD): a systematic review and meta-analysis

Supplementary Materials

1. The search strategy of the systematic review

Ovid MEDLINE

[Pulmonary Disease, Chronic Obstructive. or COPD.tw. or chronic obstructive pulmonary disease.tw or (chronic obstructive airway disease or coad).tw. or chronic obstructive lung disease.tw. or chronic obstructive respiratory disease.tw. or chronic airflow limitation.tw. or AECB.tw. or Bronchitis, Chronic or chronic bronchitis.tw. or Pulmonary Emphysema or emphysema.tw.] **AND** [Remote Consultation or Telemedicine or (telemedicine* or tele-medicine*).tw. or (internet* or computer* or web*).tw. or (telehealth* or tele-health*).tw or (interactive* or telecommunication*).tw. or (telephone or phone or SMS or "text messag*").tw. or (tele-monitor* or telemonitor*).tw. or (telemanagement or tele-management).tw. or (teleconsultation or tele-consultation).tw. or (telecare* or tele-care*).tw. or "telematic*".tw. or (telepharmacy or tele-pharmacy).tw. or (telenurs* or tele-nurs*).tw. or (video or email or e-mail).tw. or "remote consult*".tw. or (wireless or bluetooth).tw. or (tele-homecare or telehomecare).tw. or "remote care".tw. or (tele-support or telesupport).tw. or "mobile health*".tw. or "computer mediated therapy".tw. or (ehealth or e-health).tw. or (mhealth or m-health).tw. or Videoconferencing/ or videoconferenc*.tw. or (virtual adj3 (consult* or appointment*)).tw. or ((digital or electronic or smart or remote) adj3 (remind* or monitor*)).tw. or (electronic adj3 compliance).tw.] **AND** [medication adherence or "treatment adherence and compliance" or patient compliance or "patient acceptance of health care" or (concord* or nonconcord* or non-concord*).tw. or (complian* or noncomplian* or non-complian*).tw. or (adhere* or nonadhere* or non-adhere*).tw. or ((medication* or inhal* or drug or treatment or therapy) adj3 persist*).tw.]

Embase

[chronic obstructive lung disease or COPD.tw. or chronic obstructive pulmonary disease.tw. or (chronic obstructive airway disease or coad).tw. or chronic obstructive lung disease.tw. or chronic obstructive respiratory disease.tw. or chronic airflow limitation.tw. or AECB.tw. or chronic bronchitis or chronic bronchitis.tw. or lung emphysema or emphysema.tw.] **AND** [teleconsultation or telemedicine or (telemedicine* or tele-medicine*).tw. or (internet* or computer* or web*).tw. or (telehealth* or tele-health*).tw. or (interactive* or telecommunication*).tw. or telecommunication

or (telephone or phone or SMS or "text messag*").tw. or text messaging/ or mobile phone or (tele-monitor* or telemonitor*).tw. or (telemanagement or tele-management).tw. or (teleconsultation or tele-consultation).tw. or or (telecare* or tele-care*).tw. or "telematic*".tw. or (telepharmacy or tele-pharmacy).tw. or (telenurs* or tele-nurs*).tw. or (video or email or e-mail).tw. or e-mail or "remote consult*".tw. or (wireless or bluetooth).tw. or (tele-homecare or telehomecare).tw. or "remote care".tw. or (tele-support or telesupport).tw. or "mobile health*".tw. or "computer mediated therapy".tw. or (ehealth or e-health).tw. or (mhealth or m-health).tw. or Videoconferencing or videoconferenc*.tw. or (virtual adj3 (consult* or appointment*)).tw. or ((digital or electronic or smart or remote) adj3 (remind* or monitor*)).tw. or (electronic adj3 compliance).tw.] **AND** [(concord* or nonconcord* or non-concord*).tw. or (complian* or noncomplian* or non-complian*).tw. or (adhere* or nonadhere* or non-adhere*).tw. or medication compliance or patient compliance or patient attitude or ((medication* or inhal* or drug or treatment or therapy) adj3 persist*).tw.]

CINAHL

[TI emphysema OR AB emphysema OR (MH "Emphysema") OR TI chronic bronchitis OR AB chronic bronchitis OR (MH "Bronchitis, Chronic") OR TI AECB OR AB AECB OR TI chronic airflow limitation OR AB chronic airflow limitation OR TI chronic obstructive respiratory disease OR AB chronic obstructive respiratory disease OR TI chronic obstructive lung disease OR AB chronic obstructive lung disease OR TI ((chronic obstructive airway disease) or coad) OR AB ((chronic obstructive airway disease) or coad) OR TI chronic obstructive pulmonary disease OR AB chronic obstructive pulmonary disease OR TI COPD OR AB COPD OR (MH "Pulmonary Disease, Chronic Obstructive")] **AND** [TI (electronic n3 compliance) OR AB (electronic n3 compliance) OR TI (((digital or electronic or smart or remote) n3 (remind* or monitor*))) OR AB (((digital or electronic or smart or remote) n3 (remind* or monitor*))) OR TI ((virtual n3 (consult* or appointment*))) OR AB ((virtual m3 (consult* or appointment*))) OR AB videoconferenc* OR TI videoconferenc* OR (MH "Telenursing") OR (MH "Videoconferencing") OR TI (mhealth or m-health) OR AB (mhealth or m-health) OR TI (ehealth or e-health) OR AB (ehealth or e-health) OR TI "computer mediated therapy" OR AB "computer mediated therapy" OR TI "mobile health*" OR AB "mobile health*" OR TI (tele-support or telesupport) OR AB (tele-support or telesupport) OR TI "remote care" OR AB "remote care" OR TI (tele-homecare or telehomecare) OR AB (tele-homecare or telehomecare) OR TI (wireless or bluetooth) OR AB (wireless or bluetooth) OR TI "remote consult*" OR AB "remote consult*" OR TI (video or email or e-mail) OR AB (video or email or e-mail) OR TI (telenurs* or tele-nurs*) OR AB (telenurs* or tele-nurs*) OR TI (telepharmacy or tele-pharmacy) OR AB (telepharmacy or tele-pharmacy) OR TI "telematic*" OR AB "telematic*"

TI (telecare* or tele-care*) OR AB (telecare* or tele-care*)] **AND** [TI (((medication* or inhal* or drug or treatment or therapy) n3 persist*)) AND AB (((medication* or inhal* or drug or treatment or therapy) n3 persist*)) OR TI (adhere* or nonadhere* or non-adhere*) OR AB (adhere* or nonadhere* or non-adhere*) OR TI (complian* or noncomplian* or non-complian*) OR AB (complian* or noncomplian* or non-complian*) OR AB (concord* or nonconcord* or non-concord*) OR TI (concord* or nonconcord* or non-concord*) OR (MH "Patient Compliance") OR (MH "Medication Compliance")]

Cochrane CENTRAL

[MeSH descriptor: [Pulmonary Disease, Chronic Obstructive] explode all trees IR (COPD): ti,ab,kw or (chronic obstructive pulmonary disease): ti,ab,kw or ((chronic obstructive airway disease) or coad): ti,ab,kw or (chronic obstructive lung disease): ti,ab,kw or (chronic obstructive respiratory disease): ti,ab,kw or (chronic airflow limitation): ti,ab,kw or (AECB): ti,ab,kw or MeSH descriptor: [Bronchitis, Chronic] explode all trees or (chronic bronchitis): ti,ab,kw or MeSH descriptor: [Pulmonary Emphysema] explode all trees or (emphysema): ti,ab,kw] **AND** [MeSH descriptor: [Remote Consultation] explode all trees or MeSH descriptor: [Telemedicine] explode all trees or (telemedicine* or tele-medicine*):ti,ab,kw or (internet* or computer* or web*):ti,ab,kw or (telehealth* or tele-health*):ti,ab,kw or (interactive* or telecommunication*):ti,ab,kw or (telephone or phone or SMS or "text messag*"):ti,ab,kw or (tele-monitor* or telemonitor*):ti,ab,kw or (telemanagement or tele-management):ti,ab,kw or (teleconsultation or tele-consultation):ti,ab,kw or (telecare* or tele-care*):ti,ab,kw or ("telematic*"):ti,ab,kw or (telepharmacy or tele-pharmacy):ti,ab,kw or (telenurs* or tele-nurs*):ti,ab,kw or (video or email or e-mail):ti,ab,kw or ("remote consult*"):ti,ab,kw or (wireless or bluetooth):ti,ab,kw or (tele-homecare or telehomecare):ti,ab,kw or ("remote care"):ti,ab,kw or (tele-support or telesupport):ti,ab,kw or ("mobile health*"):ti,ab,kw or ("computer mediated therapy"):ti,ab,kw or (ehealth or e-health):ti,ab,kw or (mhealth or m-health):ti,ab,kw or MeSH descriptor: [Videoconferencing] explode all trees or (videoconferenc*):ti,ab,kw or (virtual near/3 (consult* or appointment*)):ti,ab,kw or ((digital or electronic or smart or remote) near/3 (remind* or monitor*)):ti,ab,kw or (electronic near/3 compliance):ti,ab,kw] **AND** [MeSH descriptor: [Medication Adherence] explode all trees or (((medication* or inhal* or drug or treatment or therapy) near/3 persist*)):ti,ab,kw]

Figure A: ***Panel (A):*** *Risk of bias assessment for randomised controlled trials (RoB2)* ***Panel (B):*** *Risk of bias assessment for non-randomised studies (ROBINS-I)*

(A)

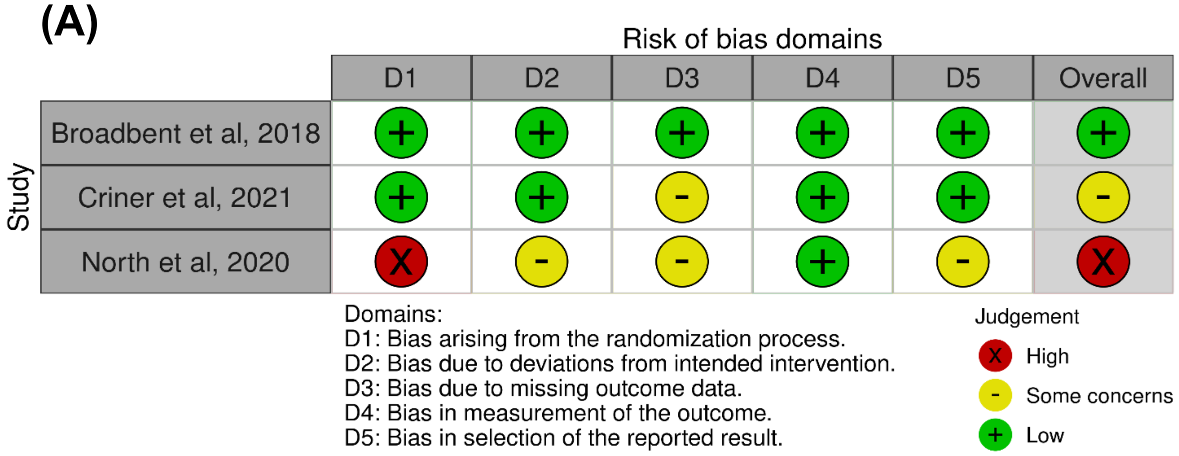
Risk of bias domains
D1 D2 D3 D4 D5 Overall
Study
Broadbent et al, 2018: + + + + + +
Criner et al, 2021: + + - + + -
North et al, 2020: X - - + - X
Domains:
D1: Bias arising from the randomization process.
D2: Bias due to deviations from intended intervention.
D3: Bias due to missing outcome data.
D4: Bias in measurement of the outcome.
D5: Bias in selection of the reported result.
Judgement
X High
- Some concerns
+ Low

(B)

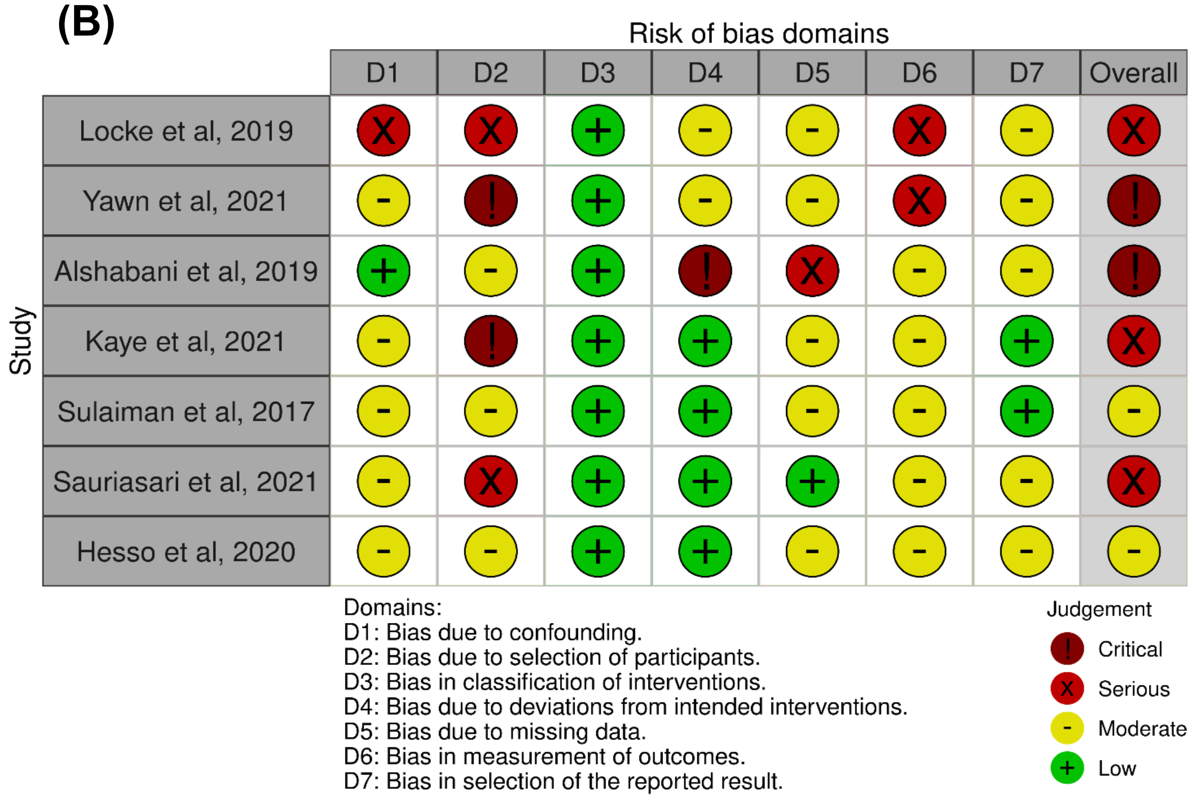
Risk of bias domains
D1 D2 D3 D4 D5 D6 D7 Overall
Study
Locke et al, 2019: X X + - - X - X
Yawn et al, 2021: - ! + - - X - !
Alshabani et al, 2019: + - + ! X - - !
Kaye et al, 2021: - ! + + - - + X
Sulaiman et al, 2017: - - + + - - + -
Sauriasari et al, 2021: - X + + + - - X
Hesso et al, 2020: - - + + - - - -
Domains:
D1: Bias due to confounding.
D2: Bias due to selection of participants.
D3: Bias in classification of interventions.
D4: Bias due to deviations from intended interventions.
D5: Bias due to missing data.
D6: Bias in measurement of outcomes.
D7: Bias in selection of the reported result.
Judgement
! Critical
X Serious
- Moderate
+ Low

Figure B: *Funnel plot for RCTs comparing the mean change in maintenance inhaler adherence rate* ***(Panel A)*** *and Clinical COPD Questionnaires (CCQ) scores* ***(Panel B)*** *between digitalized interventions delivered by healthcare providers with full features of digital platforms versus standalone passive EIM among people with COPD.*

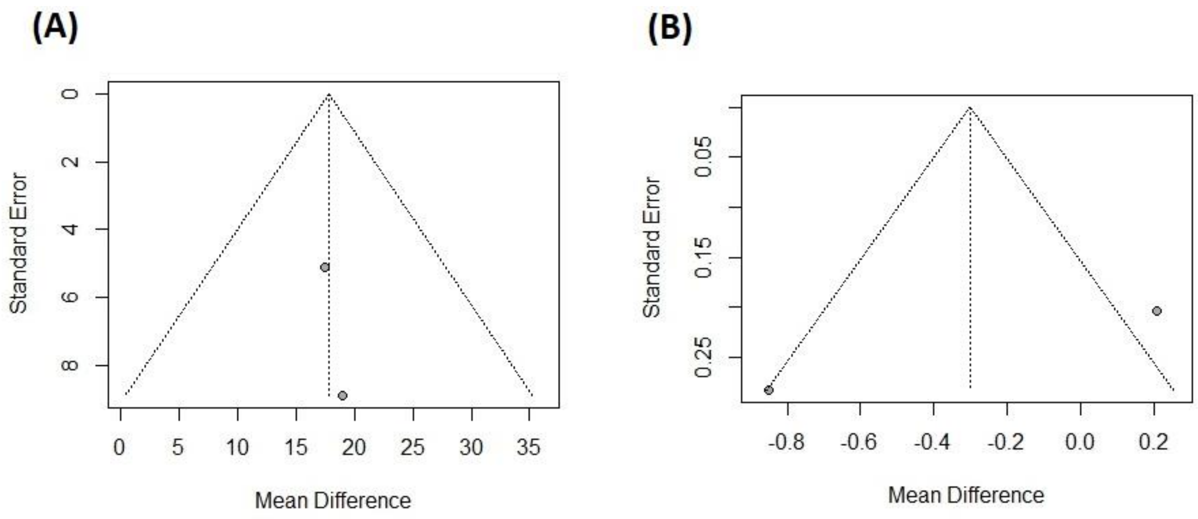

Figure C: *Funnel plot for the change in mean COPD Assessment Test (CAT) scores among people with COPD after digital interventions on maintenance inhaler adherence*

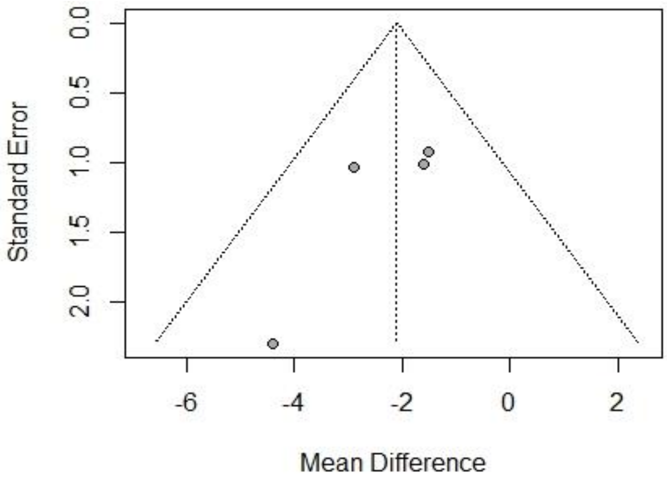

Figure D: *Funnel plot from the proportional meta-analysis of observation studies reporting participants experienced technical issues*

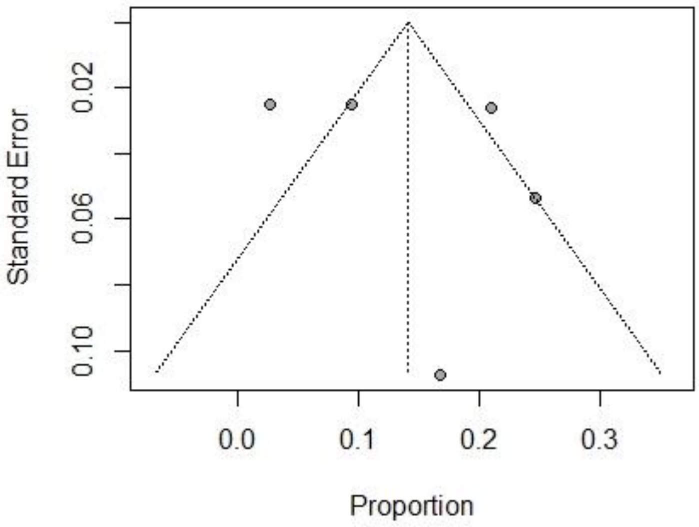

Figure E: *Forest and funnel plots from the proportional meta-analysis of observation studies reporting the percentage of participants who found digital platforms convenient to use*

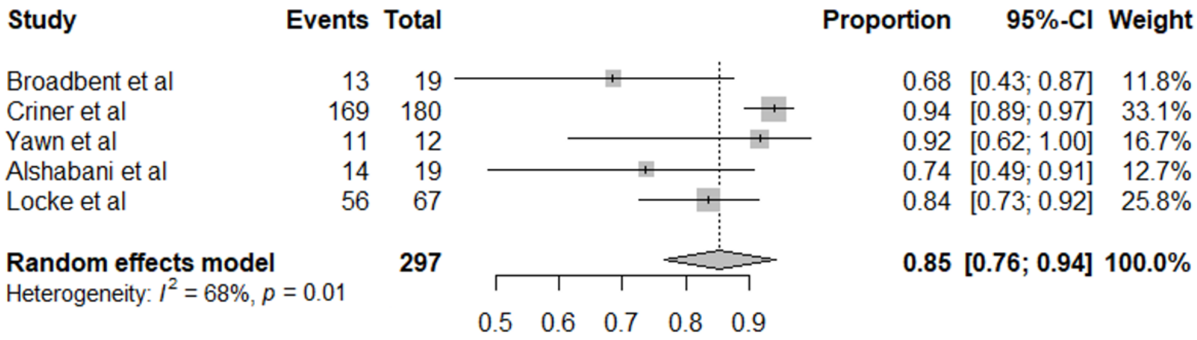

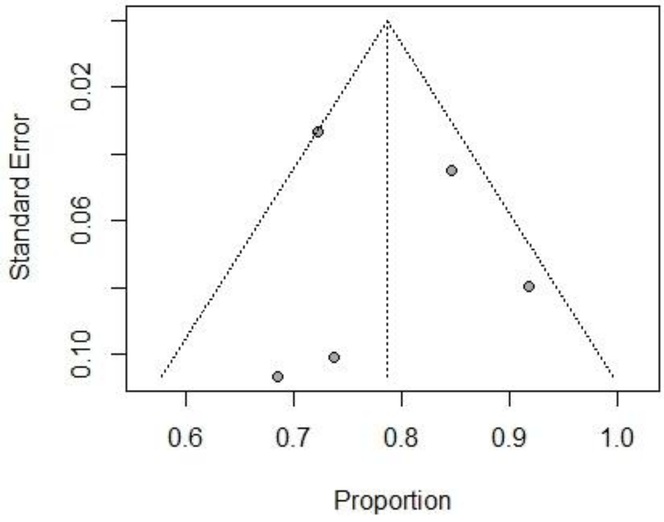

Figure F: *Forest and funnel plots from the proportional meta-analysis of observation studies reporting the percentage of participants who perceived reminders from digital platforms as useful*

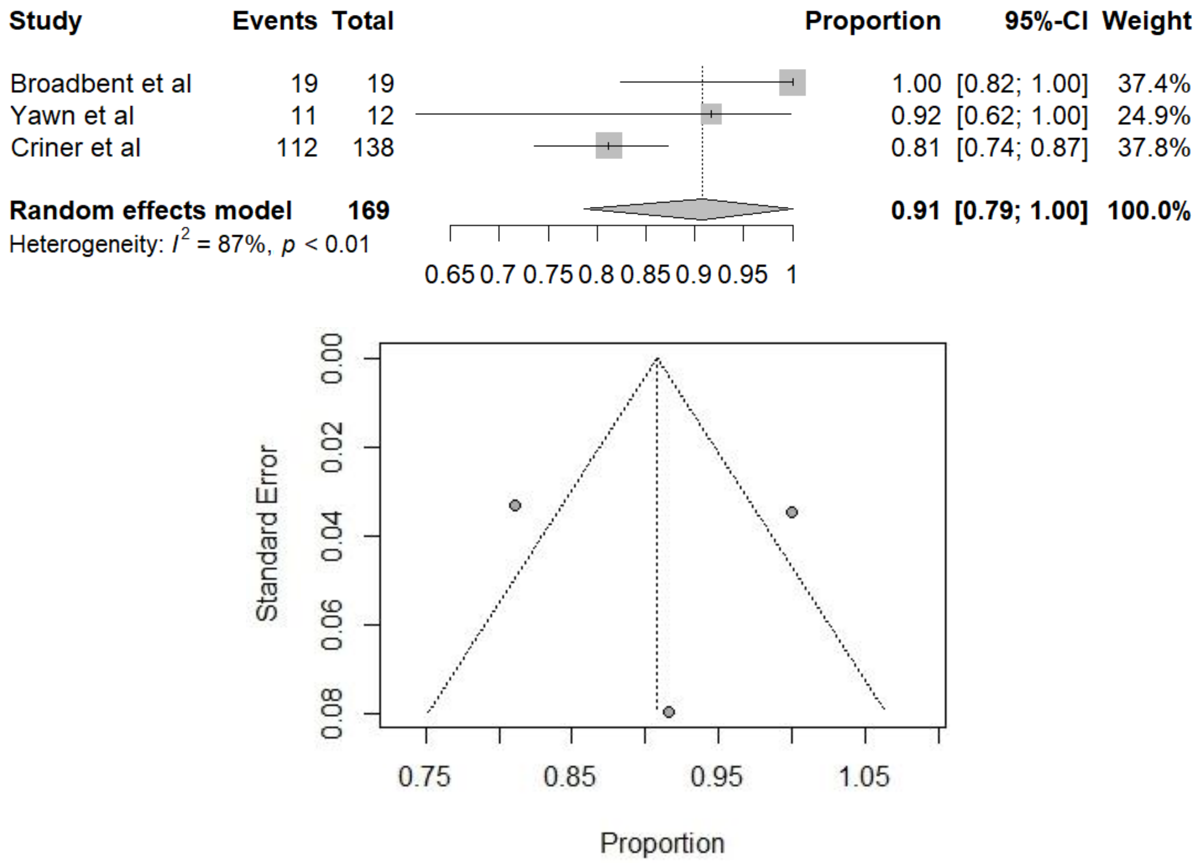

Figure G: *Forest and funnel plots from the proportional meta-analysis of observation studies reporting the attrition rate.*

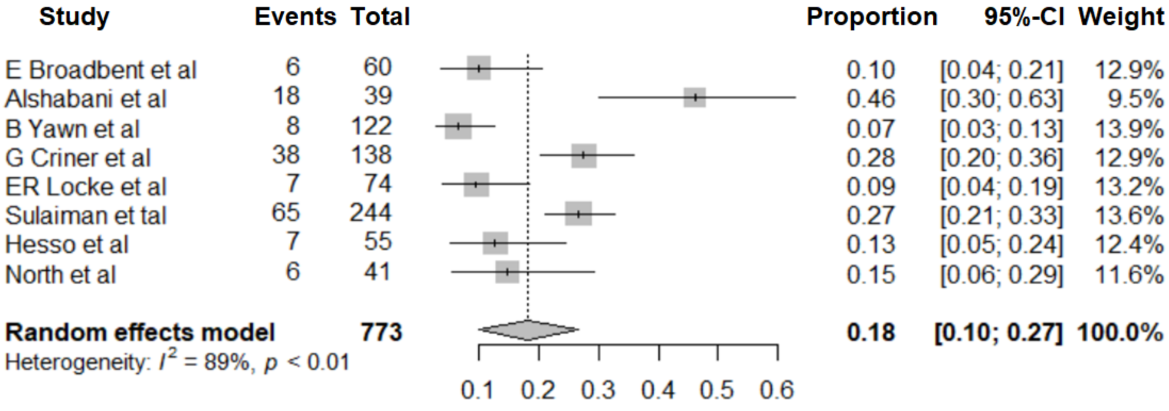

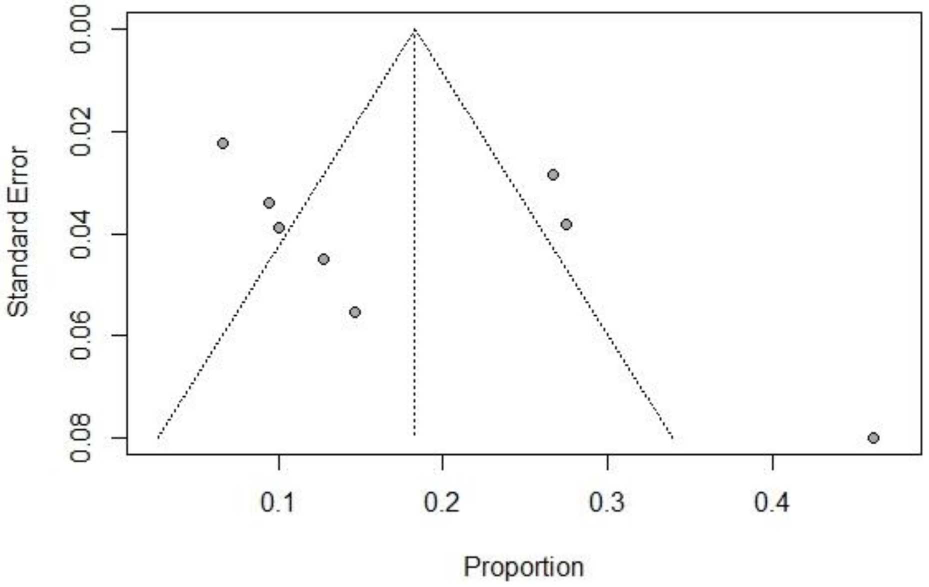
Standard Error
0.00
0.02
0.04
0.06
0.08
0.1
0.2
0.3
0.4
Proportion

Table S1: Digital platform designs that were employed among the studies investigating maintenance inhaler adherence in COPD

Study ID	Digital platform and tools	Major features and settings of the study apps	Additional intervention by healthcare providers	Study population characteristics
**Alshabani et al[1]**	Propeller Health (USA) (Digital inhalers paired with mobile App)	- Tracking date and time of actuation<br>- AV reminders (no controller uses for 4 consecutive days or higher reliever use ≥ 1.64 times (SD) above the average)	A phone call by the study team with alerts	- Adult spirometry-proven COPD patients<br>- ≥1 ED visit or hospitalisation in the prior year
**Yawn et al[2]**	Propeller Health (USA) (Digital inhalers paired with mobile App)	- Tracking date and time of actuation<br>- AV reminders (no controller uses for 4 consecutive days or higher reliever use above the average	None	- Self-reported COPD patients who were using ELLIPTA inhalers and smartphones.<br>- residents of USA
**Kaye et al[3]**	Propeller Health (USA) (Digital inhalers paired with mobile App)	- Tracking date and time of actuation<br>- Schedule-based reminders<br>- Feedback on inhaler use trends<br>- Education content on COPD	Additional gamified features if adherence was resistant to all app-derived measures.	- COPD diagnosis<br>- residents of USA<br>- Controllers compatible with EMM
**Broadbent et al[4]**	iRobi robot (Yujin Robot Limited, Korea)<br>Adherium's Hailie® devices	- Tracking date and time of actuation<br>- Scheduled inhaler reminders and rehabilitation<br>- Feedback on health status and inhaler use trends<br>- Measure oxygen level, FEV1, and HR<br>- Education modules on inhaler use and rehabilitation exercise	"I am feeling unwell" function on demand (i.e., observations done by robot and advice given to call emergency or GP and alert sent to study team).<br>Direct contact from the study team with inhaler adherence alerts	- Severe or Very severe COPD diagnosis<br>- Previous admission in the past year<br>- Housebound (going out < 4 times/ week)<br>- Living alone or with a spouse who is largely housebound<br>- Rural residents with poor social support
**Criner et al[5]**	BreatheMate digital inhalers attached to budesonide/formoterol pMDI	- Tracking data-and-time stamps of inhaler use<br>- pre-emptive as well as missed dose reminders<br>- Monthly reminders to obtain refills, overuse alerts (>10 puffs/day of controllers)	Direct contact from the study team with inhaler overuse alerts	- Adult moderate-very severe COPD patients<br>- ≥ 10 pack-year smoking history

				- ICS/LABA controller for at least 3 months of use
**Hesso et al[6]**	INCA™ attached to salmeterol/fluticasone Diskus inhaler	- Collect acoustic recordings of inhalations and provide the date, time, and quality of inhaler actuation	None	- Adult COPD and asthma patients<br>- Established on salmeterol/fluticasone DPI for at least 6 months
**Sulaiman et al[7]**	INCA™ attached to salmeterol/fluticasone Diskus inhaler	- Collect acoustic recordings of inhalations and provide the date, time, and quality of inhaler actuation	None	- Adult spirometric-proven COPD patients following admission with or without association with exacerbation<br>- History of smoking and previous exacerbation in the past year<br>- Established on salmeterol/fluticasone DPI
**North et al[8]**	myCOPD app (multi-faceted education app) in the intervention arm	- App features (multi-faceted education programs including weekly pre-recorded inhaler training videos, and environmental alerts)	None	- Adult COPD patients following an admission to hospital due to acute exacerbation of COPD
**Sauriasari et al[9]**	Pre-recorded online training sessions sent via WhatsApp application	- Pre-recorded online training video (<2mins) detailing inhalation technique		- Adult COPD patients diagnosed 1 month before the study, active WhatsApp number to contact researchers, maintenance inhaler use.
**Locke et al[10]**	Software (Cisco Jabber Video for Telepresence 4.5) and a webcam provided to patients	- Live monthly inhaler training sessions by pharmacists		- Rural COPD (93%) and asthma patients

Abbreviations: INCA™ (Inhaler Compliance Assessment device), AV (Audio-visual), FEV1 (Forced Expiratory Volume in 1 second), HR (Heart Rate),pMDI (pressurised metered-dose inhaler), DPI (dry powder inhaler)

Table S2: Summary of findings according to the GRADE approach

Patient or population: Adult moderate-severe COPD patients on maintenance inhaler therapy

Setting: Outpatient, Secondary care

Intervention: digitalised inhaler adherence intervention

Outcomes	Impact	No of participants (Studies)	Certainty of the evidence (GRADE)	Comments
Comparison of maintenance inhaler adherence rate between the intervention by healthcare providers (HCPs) alongside EIM with full features of digital platform (treatment group) Vs passive EIM only (comparator)	Adherence rate (AR), mean (SD)<br>- Broadbent et al[4]: 48.5% (34.1) versus 29.5% (32.4) between treatment and comparator group, p=0.03.<br>- Criner et al[5]: 77.6% vs 60.2%, p <0.001 (treatment group Vs comparator group)<br>**Meta-analysis: 17.8% (95% CI: 9.1-26.5, I^2=0%)**	156 (2 RCTs)	⊕⊕⊕◯<br>Moderate[a,b]	Interventions involving HCPs and digital adherence aids may increase the maintenance inhaler adherence compared to passive adherence monitoring without reminders
Change in CCQ (symptom) scores compared between the intervention by healthcare providers (HCPs) alongside EIM with full features of digital platform (treatment group) Vs passive EIM only (comparator)	- Broadbent et al[4]: mean (SD): 3.61 (1.07) Vs 2.76 (1.01) between treatment and control group.<br>- Criner et al[5]: mean (SD): 2.18 (0.82) Vs 2.39 (1.17), p=0.80 between treatment and control group.<br>**Meta-analysis of mean difference (MD): -0.3 (95% CI: -1.34 - 0.74, I^2=89%)**	198 (2 RCTs)	⊕⊕⊕◯<br>Moderate[a]	No significant additional improvement in HRQoL assessed by the mean difference in CCQ scores between the two intervention approaches.
The impact of digitalised intervention of maintenance inhaler uses on COPD-related healthcare utilization (HCU)	- North et al[8]: Odds of readmissions, OR=0.38 (95%CI: 0.07- 1.99) between remote inhaler technique training and standard of care.<br>- Alshabani et al[1]: mean (SD) COPD-related HCU per year, pre (3.4 (SD 3.2) to post 2.2 (SD 2.3) , p = 0.01].	154 (3 studies)	⊕◯◯◯<br>Very low[a,c,d]	Digitalised interventions on maintenance inhaler use may reduce COPD-related HCU.

	COPD-related hospitalisations, [pre 2.3 (2.1) Vs post 1.6 (2.0), p = 0.04]. ED visits, [pre1.1 (1.5) Vs post 0.5 (0.8), p = 0.02] after the intervention.<br><br>- Locke et al[10]: No significant difference in ED visits or hospitalisations post-intervention p > 0.05.			
The impact of digitalised interventions on maintenance inhaler uses on exacerbation rate of COPD	- North et al[8]: Relative risk of exacerbation, RR = 0.58 (95%CI:0.32-1.04) between treatment and control arms<br><br>- Locke et al[10]: 16 events in 11 participants 6 months before and 9 events in 8 participants 6 months after the intervention.<br>- Alshabani et al[1]: No change in mean (SD) rescue pack usage after intervention [pre 1.9(1.9) Vs post 1.8(2.4), p = 0.34].	115 (2 studies)	⊕◯◯◯<br>Very low[a,b,c,d]	Digitalised interventions on maintenance inhaler use may have little or no impact on the exacerbation rate of COPD.
The impact on HRQoL assessed by CAT score by digitalised interventions on maintenance inhaler use	- North et al[8]: Mean difference MD(SD) by 4.49 (95% CI: -8.41 to -0.58) in the treatment arm compared to standard of care<br>- Sauriasari et al[9]: MD (SD) = 2 (95%CI,1-3) after intervention<br>- Yawn et al[2]: MD (SD) = 0.83 (95%CI, -1.15-2.81) after intervention<br>**Meta-analysis of MD = -1.9 (95%CI, -3.0; -0.8, I^2=12%)**	165 (3 studies)	⊕◯◯◯<br>Very low[a,b,c,d]	Digitalised interventions on maintenance inhaler use improved the CAT score but did not reach MCID[11].
**GRADE Working Group grades of evidence**<br>High certainty: we are very confident that the true effect lies close to that of the estimate of the effect.<br>Moderate certainty: we are moderately confident in the effect estimate: the true effect is likely to be close to the estimate of the effect, but there is a possibility that it is substantially different.				

Low certainty: our confidence in the effect estimate is limited: the true effect may be substantially different from the estimate of the effect.<br>Very low certainty: we have very little confidence in the effect estimate: the true effect is likely to be substantially different from the estimate of effect.
**Explanations**<br>a. Recruitment was limited to patients using specific maintenance inhalers compatible with EMM<br>b. Wide range of confidence interval<br>C. Downgraded as the analysis included cohort studies<br>d. Bias assessment deemed high risk due to missing outcome data<br><br>**Abbreviations:** CAT = COPD Assessment Test, HRQoL = Health-related quality of life, CCQ score = Clinical COPD Questionnaire, MCID = Minimal clinically important difference

References

1. Alshabani K, Attaway AA, Smith MJ, et al. Electronic inhaler monitoring and healthcare utilization in chronic obstructive pulmonary disease. *J Telemed Telecare*. 2020; 26: 495-503.

2. Yawn BP, McCreary GM, Linnell JA, et al. Pilot Study of a Patient Experience with an ELLIPTA Inhaler Electronic Medication Monitor and Associated Integrated System: A Prospective Observational Study Using the COPD Patient-Powered Research Network. *Chronic Obstr Pulm Dis*. 2021; 8: 488-501.

3. Kaye L, Gondalia R, Thompson A, Stempel DA and Barrett MA. The relationship between objective app engagement and medication adherence in asthma and COPD: a retrospective analysis. *Scientific Reports*. 2021; 11: 24343.

4. Broadbent E, Garrett J, Jepsen N, et al. Using Robots at Home to Support Patients With Chronic Obstructive Pulmonary Disease: Pilot Randomized Controlled Trial. *J Med Internet Res*. 2018; 20: e45.

5. Criner GJ, Cole T, Hahn KA, Kastango K, Eudicone J and Gilbert I. The Impact of Budesonide/Formoterol pMDI Medication Reminders on Adherence in Chronic Obstructive Pulmonary Disease (COPD) Patients: Results of a Randomized, Phase 4, Clinical Study. *Int J Chron Obstruct Pulmon Dis*. 2021; 16: 563-77.

6. Hesso I, Nabhani Gebara S, Greene G, Co stello RW and Kayyali R. A quantitative evaluation of adherence and inhalation technique among respiratory patients: An observational study using an electronic inhaler assessment device. *International Journal of Clinical Practice*. 2020; 74: e13437.

7. Sulaiman I, Cushen B, Greene G, et al. Objective Assessment of Adherence to Inhalers by Patients with Chronic Obstructive Pulmonary Disease. *Am J Respir Crit Care Med*. 2017; 195: 1333-43.

8. North M, Bourne S, Green B, et al. A randomised controlled feasibility trial of E-health application supported care vs usual care after exacerbation of COPD: the RESCUE trial. *NPJ Digit Med*. 2020; 3: 145.

9. Sauriasari R, Madani RA, Rozaliyani A and Sudiana D. The effect of repeated education using live demonstrations and videos of how to use inhalation drugs on quality of life for COPD patients. *Heliyon*. 2021; 7: e07870.

10. Locke ER, Thomas RM, Woo DM, et al. Using Video Telehealth to Facilitate Inhaler Training in Rural Patients with Obstructive Lung Disease. *Telemed J E Health*. 2019; 25: 230-6.

11. Kon SS, Canavan JL, Jones SE, et al. Minimum clinically important difference for the COPD Assessment Test: a prospective analysis. *Lancet Respir Med*. 2014; 2: 195-203.
